# Effects of Alzheimer’s and Vascular Pathologies on Structural Connectivity in Early- and Late-Onset Alzheimer’s Disease

**DOI:** 10.3389/fnins.2021.606600

**Published:** 2021-02-16

**Authors:** Wha Jin Lee, Cindy W. Yoon, Sung-Woo Kim, Hye Jin Jeong, Seongho Seo, Duk L. Na, Young Noh, Joon-Kyung Seong

**Affiliations:** ^1^School of Biomedical Engineering, Korea University, Seoul, South Korea; ^2^Department of Neurology, School of Medicine, Inha University, Incheon, South Korea; ^3^Neuroscience Research Institute, Gachon University, Incheon, South Korea; ^4^Department of Neuroscience, College of Medicine, Gachon University, Incheon, South Korea; ^5^Department of Electronic Engineering, Pai Chai University, Daejeon, South Korea; ^6^Department of Neurology, Samsung Medical Center, School of Medicine, Sungkyunkwan University, Seoul, South Korea; ^7^Neuroscience Center, Samsung Medical Center, Seoul, South Korea; ^8^Department of Neurology, Gil Medical Center, College of Medicine, Gachon University, Incheon, South Korea; ^9^Department of Health Sciences and Technology, Gachon Advanced Institute for Health Sciences & Technology (GAIHST), Gachon University, Incheon, South Korea; ^10^Department of Artificial Intelligence, Korea University, Seoul, South Korea; ^11^Interdisciplinary Program in Precision Public Health, Korea University, Seoul, South Korea

**Keywords:** early-onset AD, late-onset AD, positron emission tomography, tau, amyloid, small vessel disease, white matter connectivity

## Abstract

Early- and late-onset Alzheimer’s disease (AD) patients often exhibit distinct features. We sought to compare overall white matter connectivity and evaluate the pathological factors (amyloid, tau, and vascular pathologies) that affect the disruption of connectivity in these two groups. A total of 50 early- and 38 late-onset AD patients, as well as age-matched cognitively normal participants, were enrolled and underwent diffusion-weighted magnetic resonance imaging to construct fractional anisotropy-weighted white matter connectivity maps. [^18^F]-THK5351 PET, [^18^F]-Flutemetamol PET, and magnetic resonance imaging were used for the evaluation of tau and related astrogliosis, amyloid, and small vessel disease markers (lacunes and white matter hyperintensities). Cluster-based statistics was performed for connectivity comparisons and correlation analysis between connectivity disruption and the pathological markers. Both patient groups exhibited significantly disrupted connectivity compared to their control counterparts with distinct patterns. Only THK retention was related to connectivity disruption in early-onset AD patients, and this disruption showed correlations with most cognitive scores, while late-onset AD patients had disrupted connectivity correlated with amyloid deposition, white matter hyperintensities, and lacunes in which only a few cognitive scores showed associations. These findings suggest that the pathogenesis of connectivity disruption and its effects on cognition are distinct between EOAD and LOAD.

## Introduction

Alzheimer’s disease (AD) is the most common type of neurodegenerative disease, marked by amyloid plaques and neurofibrillary tangles present in gray matter (Blennow et al., 2006; Bastin and Salmon, 2014). Age is known to be associated with AD prevalence, with the elderly being at higher risk. However, there exists a small proportion of AD patients for whom disease symptoms emerge at a relatively younger age, often with severe socioeconomic implications. Several studies have investigated the relationship between age and AD and have identified differences in the clinical features that exist between early-onset AD (EOAD) and late-onset AD (LOAD), distinguished by the onset age of 65. From a neuropsychological perspective, memory function is generally more disrupted in LOAD patients (Jacobs et al., 1994; Koss et al., 1996; Smits et al., 2012), while EOAD patients exhibit a wider range of cognitive dysfunction, which can also include non-memory cognitive domains (Fujimori et al., 1998; Kaiser et al., 2012; Smits et al., 2012).

Significant research efforts have focused on identifying the factors associated with cognitive heterogeneities between EOAD and LOAD. Some studies have revealed that the distribution patterns of pathologies, patterns, or the rate of brain atrophy in EOAD are distinct from that of LOAD. EOAD patients tend to show widespread cortical atrophy even in the early stage, especially in the posterior parietal and lateral temporal cortices, with relatively less involvement of the medial temporal structures (Frisoni et al., 2005, 2007; Canu et al., 2012; Cho et al., 2013a). Regarding AD pathology, EOAD patients have shown a greater extent of neuritic plaque and neurofibrillary tangle burden in the frontal and parietal lobes than LOAD patients in postmortem studies (Marshall et al., 2007). In amyloid PET studies, EOAD patients have exhibited higher amyloid burden in the parietal cortex (Ossenkoppele et al., 2012), basal ganglia, and thalamus (Cho et al., 2013b). Recent tau PET studies with [^18^F]flortaucipir showed that EOAD had greater tau burden in the neocortex compared to LOAD patients (Cho et al., 2017; Schöll et al., 2017). [^18^F]-THK5351 PET also showed greater retention in the association cortex including prefrontal, parietal, precuneus, and posterior cingulate cortex in EOAD patients compared to LOAD (Noh et al., 2017). Vascular pathology is also known to be part of the multifactorial mechanisms present in LOAD (Ortner et al., 2015; Iturria-Medina et al., 2016).

Brain connectivity is also considered to be one of the key factors explaining the clinical heterogeneities between EOAD and LOAD. A recent study has shown that there exist different patterns of damage in terms of the interhemispheric structural and functional connectivity of EOAD and LOAD, and these patterns may lead to distinct clinical profiles (Li et al., 2018). As previously reported, the spatial atrophy pattern of neurodegenerative diseases resembles a distinct intrinsic functional connectivity (Buckner et al., 2005; Seeley et al., 2009), and network-based analysis of brain white matter (WM) connections can reveal the structural basis of cognitive dysfunction in AD (Reijmer et al., 2013), which implies the importance of brain network in the progression of AD. Therefore, clarifying how various pathologies are related with alterations of the brain connectivity may enhance understanding of progressive mechanisms in AD continuum, which is necessary to assess future therapeutic strategies of the disease (Wattmo et al., 2013; Hanseeuw et al., 2019). In the same manner, several research groups have investigated about relationships between the aforementioned pathological markers and brain connectivity. Diffusion tensor imaging (DTI) metrics or structural connectome alterations showed correlations with amyloid burden (Racine et al., 2014) or levels of cerebrospinal fluid amyloid and tau (Tucholka et al., 2018). Higher vascular burden represented by white matter hyperintensities (WMHs) also have been shown to be associated with a reduced functional connectome in AD (Taylor et al., 2017). However, these previous studies addressed only single pathological factor or could not demonstrate associations with brain-wide regional burden of these factors using *in vivo* positron emission tomography (PET) imaging. To date, above all, there have been no previous studies considering relationship between pathological markers and WM structural network to explain clinical heterogeneities between EOAD and LOAD.

In this study, we sought to assess the relationship between AD pathologies (tau and amyloid) or small vessel disease (SVD) markers (lacunes and WMH) and WM structural connectivity in patients with EOAD and LOAD. We used [^18^F]-THK5351 (THK) as a tracer of tau and reactive astrogliosis and [^18^F]-Flutemetamol (FLUTE) as a tracer of amyloid. THK is known to trace not only neurofibrillary tangles but a combination of neurofibrillary tangles and astrocytosis (Ng et al., 2017; Harada et al., 2018). We identified disrupted patterns of connectivity in whole brain for EOAD and LOAD compared to their age-matched controls and derived a set of interregional connections whose degrees of damage, specified by *W* scores, were highly related to the amount of each pathological marker. Permutation-based network-based statistics (NBS) (Zalesky et al., 2010) and cluster-based statistics (CBS) (Han et al., 2013) were performed for each process, which were proposed to cope with the problem of multiple comparison while preventing too conservative correction in connectivity analysis using group differences or correlation coefficients, respectively. We hypothesize that each pathology is distinctly related to disrupted pattern of WM structural connectivity for EOAD and LOAD, and the different pathology-related connectivity disruptions between EOAD and LOAD might be associated with the disparate clinical features of them.

## Materials and Methods

### Participants

We recruited 93 clinically diagnosed AD dementia patients and 66 cognitively normal (CN) subjects at Gachon University Gil Medical Center in the Republic of Korea. All subjects received scans at Gil Medical Center from October 2015 to June 2017. Of the 159 subjects, we excluded one CN subject because their b-value and b-vector data could not be extracted, as well as one AD patient due to an incorrect entry for the diffusion-weighted magnetic resonance imaging (MRI) volumes (dicom), one AD patient due to a file conversion error, and three AD patients due to fiber tracking errors. Ultimately, 88 AD patients and 65 CN subjects were assessed in the study.

All AD patients fulfilled the probable AD criteria as proposed by the National Institute of Neurological and Communicative Disorders and Stroke, and the AD and Related Disorders Association (NINCDS-ADRDA) (McKhann et al., 1984). Early-onset AD was defined when symptoms had developed before age 65 and late-onset AD when symptoms had developed after the age of 65. All patients completed a clinical interview and underwent a standardized neuropsychological examination. Detailed information on the test items is in Supplementary Table 1. Among the AD patients, five patients were diagnosed with focal variants of AD. One patient met the criteria for posterior cortical atrophy (PCA) (Crutch et al., 2012); three patients were diagnosed with logopenic-variant primary progressive aphasia (LPA) (Gorno-Tempini et al., 2011), and one patient was diagnosed with a behavioral/dysexecutive variant of AD (frontal variant AD) (Ossenkoppele et al., 2015). Three patients with LPA undertook the Korean version of the Western Aphasia Battery instead of the standardized neuropsychological test due to aphasia symptoms. We excluded familial AD patients with autosomal dominant inheritance. No patients with severe WMH on MRI were included, defined as a cap or a band ≥ 10 mm wide as well as deep WMH ≥ 25 mm in length, as modified from the Fazekas ischemia criteria (Fazekas et al., 1987; Seo et al., 2009). Patients exhibiting other structural lesions on brain MRI such as territorial infarction, intracranial hemorrhage, traumatic brain injury, hydrocephalus, or WMH associated with radiation, multiple sclerosis or vasculitis were also excluded. We performed laboratory tests to rule out secondary causes of dementia addressing complete blood counts, vitamin B_12_, folate levels, thyroid function, metabolic profile, and syphili serology. Apolipoprotein E (APOE) genotype was also performed for all patients. Mini-mental State Examination (MMSE), clinical dementia rating (CDR), and clinical dementia rating sum-of-boxes (CDR SOB) results were obtained, and detailed neuropsychological function tests including attention, praxis, frontal/executive function, visual and verbal memory, language, visuoconstructive ability, and elements of Gerstmann syndrome were evaluated in all participants. Detailed items of the comprehensive test battery (Kang et al., 2003) have been described in our previous study (Lee et al., 2018).

CN subjects were recruited from volunteers in the community or spouses of patients at the Memory Disorder Clinic of Gil Medical Center (age range, 45–85; female, 45%). All CN subjects had no history of neurological/psychiatric illnesses or abnormalities detected on neurological examination. They were required to have a zero clinical dementia rating score, and normal cognitive function defined as within 1.5 standard deviations of the age- and education-corrected normative mean as determined by neuropsychological tests. There were no structural lesions including cerebral infarction, intracranial hemorrhage, traumatic brain injury, hydrocephalus, or severe WMH detected in brain MRI scans of the CN subjects. For the comparison with the EOAD or LOAD groups, CN subjects were divided into each age-matched control groups comprising 33 young controls (YC) (mean age, 57.6 years old) and 33 old controls (OC) (mean age, 75.7 years old).

Written informed consent was obtained from all participants, and the study was approved by the Institutional Review Board of Gachon University Gil Medical Center.

### Acquisition of MR Images

T1- and diffusion-weighted images of all subjects were obtained using a 3.0-Tesla MR scanner (Verio, Siemens, Erlangen, Germany) at Gil Medical Center. 3D T1 magnetization prepared rapid gradient echo (T1-MPRAGE) was acquired using the following parameters: repetition time of 1,900 ms, echo time of 2.93 ms, flip angle of 8°, pixel bandwidth of 170 Hz/pixel, matrix size of 256 × 208, 256 mm field of view, 1 number of excitations (NEX), total acquisition time of 4 min and 10 s, and 0.5 × 0.5 × 1.0 mm^3^ voxels. DTI was acquired using the following parameters: b = 0 and 900 s/mm^2^ repetition time of 12,000 ms, echo time of 78 ms, number of diffusion gradient directions = 30; flip angle of 90°, pixel bandwidth of 1,502 Hz/pixel, matrix size of 128 × 128, 256 mm field of view, 1 NEX, total acquisition time of 13 min and 26 s, and 2 × 2 × 2 mm^3^ voxels.

Other clinical MRI sequences including fluid-attenuated inversion recovery (FLAIR) and T1- and T2-weighted imaging were also acquired. The FLAIR imaging parameters used were as follows: repetition time = 9,000 ms, echo time = 122 ms, flip angle = 150°, pixel bandwidth = 287 Hz/pixel, matrix size = 256 × 224, field of view = 256 mm, NEX = 1, slice thickness = 2 mm, and total acquisition time = 2 min and 44 s. T1-weighted imaging parameters used were as follows: repetition time = 500 ms, echo time = 9.2 ms, flip angle = 70°, pixel bandwidth = 391 Hz/pixel, matrix size = 256 × 224, field of view = 256 mm, NEX = 1, slice thickness = 4 mm, total acquisition time = 3 min and 48 s. The T2-weighted imaging parameters used were as follows: repetition time = 9,650 ms, echo time = 88 ms, flip angle = 120°, pixel bandwidth = 174 Hz/pixel, matrix size = 256 × 224, field of view = 256 mm, NEX = 1, slice thickness = 4 mm, total acquisition time = 3 min and 3 s. One experienced neurologist who was blinded to other patient data reviewed the number and location of lacunes using the FLAIR and T1 and T2 images. WMH volume was calculated using the FLAIR images.

### Acquisition of PET Images

All PET scans were acquired with a Siemens Biograph 6 Truepoint PET/computed tomography (CT) scanner (Siemens, Knoxville, Tennessee, United States) using a list-mode emission acquisition. THK was synthesized and radiolabeled at Gachon University Neuroscience Research Institute, and FLUTE was purchased from Carecamp Inc. Emission scans of all subjects were processed for 20 min starting 50 min after intravenous injection (50–70 min) of 185 MBq of THK, and the subjects underwent a 20-min emission scan beginning 90 min after 185 MBq of FLUTE was injected intravenously (90–110 min). The mean intervals between THK PET and FLUTE PET scans were 10 days, and MRI scans were obtained on the same day with FLUTE PET. Attenuation correction was executed prior to all scans with low-dose CT. Individual static images were reconstructed using a 2D ordered subset expectation maximization algorithm (8 iterations and 16 subsets) and corrected for physical effects. Reconstruction was performed using the following parameters: 256 × 256 × 109 matrix; voxel size of 1.3 × 1.3 × 1.5 mm^3^.

### PET Quantification

Each THK5351 and FLUTE PET image was coregistered with the corresponding T1 image using FreeSurfer. We performed voxel-based partial volume correction (PVC) to the MRI coregistered PET images using the PETSurfer tool in FreeSurfer (Greve et al., 2014, 2016). Standardized uptake value ratios (SUVRs) were computed to address intersubject effects using cerebellar gray matter as the reference region for THK images and pons for FLUTE images. The regional SUVRs were mean values of voxels assigned to a predefined region of interest (ROI). The global retention ratio was generated based on AD-related regions including the prefrontal, superior and inferior parietal, lateral temporal, and anterior and posterior cingulate cortices (Thurfjell et al., 2014) for FLUTE images. Patients with global retention ratios > 0.62 were classified as amyloid positive (Thurfjell et al., 2014).

### White Matter Network Construction

**FIGURE 1:**
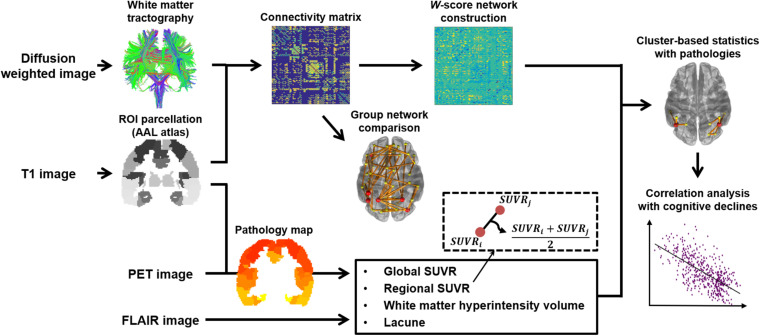
Study overview. A connectivity matrix for each group was created using T1- and diffusion-weighted images. We compared the networks between the AD groups and age-matched control groups, before *W*-score networks were generated from the AD groups with the respective reference control group. *W*-score networks were also compared and analyzed for the correlations with topological pathology maps comprising multiple factors drawn from T1 and PET images. Identified subnetworks from the correlation analyses were used to assess associations with cognitive scores.

As shown in Figure 1, white matter structural networks were constructed using DTI techniques, based on eddy-current-corrected diffusion-weighted MR images (FSL^1^). For each hemisphere, the nodes comprised 39 cortical regions of the brain defined by an automated anatomical labeling (AAL) template. Estimation of the nodes and edge strengths and detailed information on the fiber tracking process were based on our previous study (Chung et al., 2019). Specifically, the nodes of each WM structural network were extracted by using the FSL Linear Registration Tool (FLIRT) between eddy-current-corrected diffusion-weighted MR images and corresponding T1-weighted MR images and using the FSL Non-linear Registration Tool (FNIRT) between T1-weighted MR images and ICBM152 T1 templates in the MNI space where the AAL template is defined. Streamlines were acquired by whole-brain deterministic tractography between each pair of nodes, using the second order Runge–Kutta algorithm through the Diffusion toolkit (Wang et al., 2007). Fiber tracking was initiated at the eight random points of each seed voxel with a fractional anisotropy (FA) > 0.3 and ended at the voxels with FA < 0.2 or a tract turning angle of > 45°. If there existed at least one streamline between a pair of nodes, FA values were averaged across streamlines using the UCLA Multimodal Connectivity Package^2^, which referred to edge strengths (van den Heuvel and Sporns, 2011). We used FA-weighted structural networks for the statistical analysis since FA values have been considered to represent the level of microstructural organization of WM tracts (Beaulieu, 2002), known to be associated with the efficacy of the connections (Ewing-Cobbs et al., 2006; Gold et al., 2007), and therefore, the FA-weighted connectivity matrix may incorporate the WM damage better than mere streamline counts (Cho et al., 2018).

We then constructed a *W*-score network (Jack et al., 1997; La Joie et al., 2012; Ossenkoppele et al., 2015) for each WM network of AD patients to represent normalized individual disruption of WM connectivity compared to the overall white matter damage of the CN group. *W*-scores are standardized values adjusted using covariates, including age, sex, and years of education. We first created a linear regression model for each edge weight with the predictor variables in the CN group. An edge weight for the AD group can be predicted from the regression model using the variables for each patient. The *W*-score for an edge was computed as a ratio of difference between actual and predicted edge weight (namely, residual) in each AD patient to the standard deviation of residuals in the CN group. Therefore, as there is more critical connectivity disruption in AD compared to CN, the *W*-score decreases in a negative direction. It can be represented as follows:

$$W-score_{i}=\frac{E_{AD,i}-{\hat{E}}_{AD,i}}{\sqrt{Var\left(E_{CN}-{\hat{E}}_{CN}\right)}}$$

*E*_*AD,i*_ refers to an actual edge weight for the *i*th AD patient, and ${\hat{E}}_{AD,i}$ refers to a predicted value for the *i*th AD patient through the established regression model. $\sqrt{Var\left(E_{CN}-{\hat{E}}_{CN}\right)}$ is a standard deviation of residuals for the CN group. We used the YC group as a reference for the EOAD patients and OC group for the LOAD patients, respectively.

### Statistical Analyses

To compare the disrupted pattern of WM connectivity between AD and age-matched CN groups, we conducted permutation-based network-based statistics (NBS) (Zalesky et al., 2010). Edge-by-edge t-statistics were calculated for the WM connectivity of the two groups after controlling for sex and years of education. Connected components (clusters) were set with edges having higher coefficients than a given threshold. We tested the significance of each cluster size within the empirical null distribution of maximum cluster sizes estimated by 5,000 random permutations of edge weights in design matrices. *p*-values were assigned to components extracted from the original set, and 0.05 was the significance level used to determine significant clusters.

In addition, permutation-based cluster-based statistics (CBS) (Han et al., 2013) was performed to show associations between the network disruption and several pathological factors. Specifically, Pearson correlation coefficients were computed using the *W*-score network and predefined pathogenesis. In common with the previous NBS method, corrections for age, sex, and years of education were not involved. We used four factors for pathogenesis: THK retention for tau accumulation, FLUTE retention for amyloid accumulation, and lacunes and WMH volume for cerebral SVD markers. Especially in the case of regional retentions, values were averaged between each pair of ROIs to represent the local influence on an individual edge. It can take into account how the group-specific load and distribution of pathology affects each edge, while the CBS analyses using global THK and FLUTE retention values reveal global effects. We assigned *p*-values to clusters based on 5,000 random permutations of the factors and determined resultant subnetworks at the significance level of 0.05.

For comparisons of the demographic and clinical data, Wilcoxon rank sum test was conducted for continuous variables. Nominal variables were compared using chi square tests. To show relationships between the factor-related WM network disruption and neuropsychological functions, Pearson correlation coefficients were calculated using the averaged *W*-scores of the subnetwork resulting from CBS and standardized cognitive impairment scores. Multiple comparisons for scores were corrected using the Benjamini–Hochberg false discovery rate (FDR) method (Benjamini and Hochberg, 1995).

## Results

### Demographic and Clinical Characteristics

Detailed demographic and clinical information is summarized in Table 1. There were no significant differences between the EOAD and LOAD patients except for age and age at onset. Years of education and disease duration did not differ significantly in EOAD and LOAD nor did MMSE or CDR SOB. The number of APOE ε4 carriers and proportion of amyloid-positive patients was not different between the EOAD and LOAD groups.

**TABLE 1:** Demographic and clinical characteristics of the study population.

Variables	EOAD (*n* = 50)	YC (*n* = 33)	EOAD vs. YC *p*-value (statistics)	LOAD (*n* = 38)	OC (*n* = 32)	LOAD vs. OC *p*-value (statistics)	EOAD vs. LOAD *p*-value (statistics)
Age (years)	60.44 ± 5.39	57.55 ± 7.17	0.114	78.08 ± 6.63	75.66 ± 5.36	0.104	<0.001*
Age at onset (years)	57.10 ± 5.09	–	–	73.84 ± 6.12	–	–	<0.001*
Female sex, *n* (%)	34 (68.0)	13 (39.4)	0.010* (6.623)	28 (73.7)	17 (53.1)	0.074 (3.198)	0.563 (0.335)
Education (years)	9.49 ± 3.95	13.45 ± 3.52	<0.001*	7.57 ± 5.23	10.53 ± 5.32	0.028*	0.113
Disease duration (months)	39.78 ± 15.87	–	–	49.29 ± 30.88	–	–	0.384
MMSE	16.94 ± 6.02	28.76 ± 1.15	<0.001*	18.92 ± 5.88	27.03 ± 2.48	<0.001*	0.103
CDR SOB	4.87 ± 2.90	0.00 ± 0.00	<0.001*	4.77 ± 2.76	0.00 ± 0.00	<0.001*	0.971
APOE ε4 carrier, *n* (%)	25 (50.0)	8 (24.2)	0.019*(5.507)	19 (50.0)	6 (18.8)	0.007*(7.389)	1.000 (<0.001)
Amyloid positivity, *n* (%)	49 (98.0)	0 (0.0)	<0.001* (78.95)	35(92.1)	2 (6.3)	<0.001* (51.39)	0.189 (1.729)
Global FLUTE SUVR	1.07 ± 0.19	0.39 ± 0.06	<0.001*	0.87 ± 0.22	0.43 ± 0.14	<0.001*	<0.001*
Global THK SUVR	2.31 ± 0.42	1.46 ± 0.20	<0.001*	2.20 ± 0.39	1.78 ± 0.25	<0.001*	0.298
Total lacunes, median (IQR)	0 (1)	0 (1)	0.444	1 (2)	0 (2)	0.123	0.008*
Total WMH volume, median (IQR) (mm^3^)	3087.0 (3681.0)	1837.0 (1877.8)	0.004^∗^	5253.0 (6416.0)	3118.0 (4777.5)	0.059	0.007*

Data are presented as mean ± standard deviation for continuous variables and number (%) for nominal variables. Independent Wilcoxon rank sum test for continuous variables and chi square test for nominal variables. *Significant EOAD, early-onset Alzheimer’s disease; YC, young control; LOAD, late-onset Alzheimer’s disease; OC, old control; MMSE, mini-mental state examination; CDR SOB, clinical dementia rating sum-of-boxes; SUVR, standardized uptake value ratio; WMH, white matter hyperintensity.

Global THK retention did not show a significant difference between EOAD and LOAD groups (*p* = 0.298). LOAD patients had higher values for the number of lacunes and WMH volume compared to EOAD patients, whereas EOAD patients had greater values for global FLUTE retention than LOAD patients. Compared to the corresponding age-matched CN group, global THK and FLUTE retentions were significantly greater in the AD groups. In terms of SVD markers, the EOAD group only had significant WMH volume differences with the control group (*p* = 0.004), unlike LOAD (*p* = 0.059).

### Distinct Patterns of WM Connectivity Disruption in AD

**FIGURE 2:**
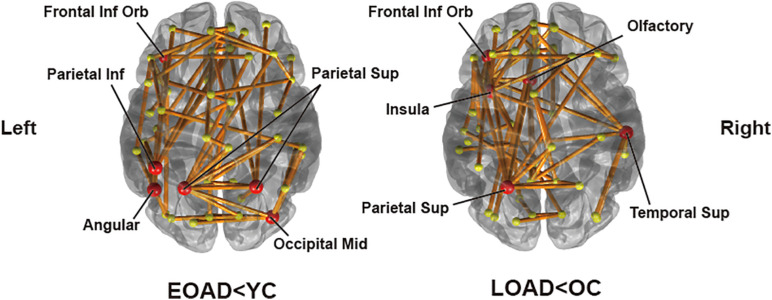
Subnetworks identified by connectivity comparisons in EOAD and LOAD with respective age-matched controls through network-based statistics. The red circle areas are representative regions, with nodal degree exceeding the mean plus standard deviation of all nodal degrees.

Significantly destructed connections between AD and CN group were identified based on group difference of edge strengths using the NBS method. A threshold for NBS was set to 2.5. A total of 68 edges had significantly lower weights in EOAD compared to YC (*p* = 0.0162). We identified the representative regions in the output subnetwork to determine the most influential regions for the distinct pattern of network disruptions, passed by the majority of disrupted edges. This included the left orbital part of the inferior frontal gyrus, right middle occipital gyrus, bilateral superior parietal lobules, left inferior parietal lobule, and left angular gyrus (Figure 2). Meanwhile, when comparing LOAD and OC, 59 edges were found to be significantly disrupted (*p* = 0.0176) with the representative regions of the left orbital part of inferior frontal gyrus, left olfactory cortex, left insula, left superior parietal lobule, and right superior temporal gyrus (Figure 2). In EOAD patients, connections linked with the parietal lobe were most vulnerable, involving 50.0% of the total edges in the subnetwork, while most damaged edges (66.1%) were related to the frontal lobe in LOAD patients.

### Correlation Between Connectivity Disruption and Pathological Markers

**FIGURE 3:**
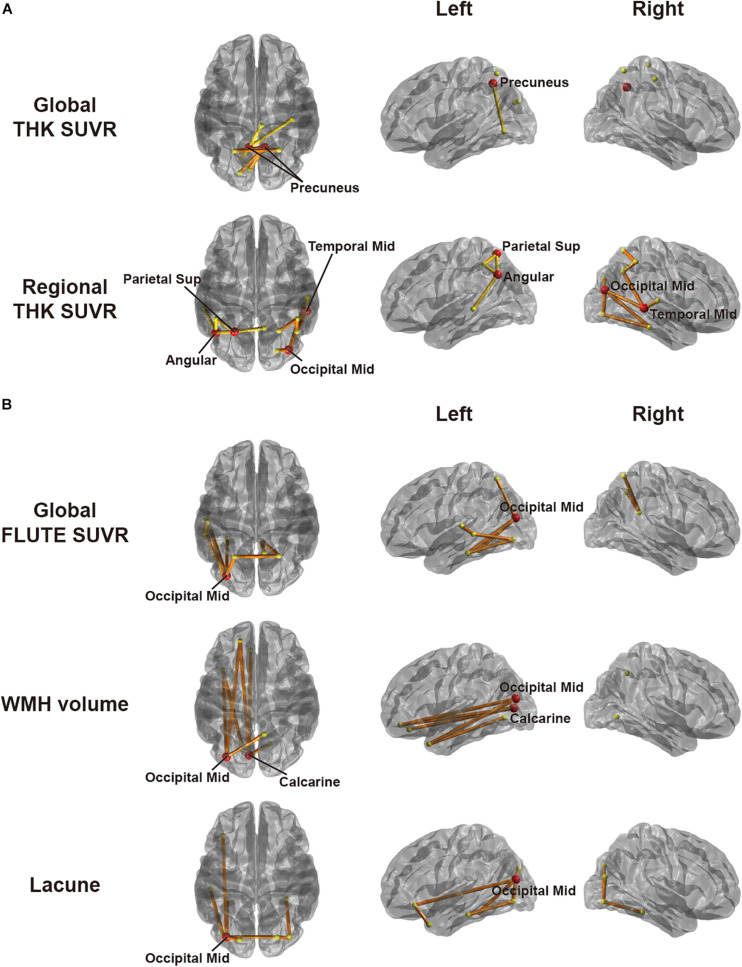
Subnetworks identified by correlation analyses between *W*-score networks and pathologies in **(A)** EOAD and **(B)** LOAD through cluster-based statistics. The factors used include global and regional THK retention in EOAD and global FLUTE retention, white matter hyperintensity volume, and lacunes in LOAD. Regional retention indicates the averaged retention between regions at both ends of an edge. The red circles are representative regions, with nodal degree exceeding the mean plus standard deviation of all nodal degrees. Edges within different clusters were displayed with different colors.

To explore associations between network disruption in each AD group and pathological markers, we specified the amount of disruption for each edge by *W*-score using respective age-matched CN group as a reference group. Figure 3 shows the results of CBS (Han et al., 2013) for correlation between the *W*-scores and each pathology using a threshold of −0.45. In EOAD, the *W*-scores had a significant negative correlation only with THK retention. Both global and regional THK retentions showed an increasing trend with greater connectivity disruptions. Detailed results are shown in Table 2. In the global view, the number of clusters was two, and the largest size was four (*p* = 0.0070). The most affected regions were identified by significantly disrupted edges affected by each pathology. The damaged edges related to global THK retention passed bilateral precuneus regions the most in EOAD (Figure 3A). With the representative regions involved in the parietal lobule, all edges in the identified subnetwork related to global THK retention were connected to the parietal lobule, which were connected to occipital regions the most (42.9%). Local values resulted in two clusters, and the size of the largest cluster was nine (*p* = 0.0004). Identified representative regions contained the right middle occipital gyrus, left superior parietal gyrus, left angular gyrus, and right middle temporal gyrus (Figure 3A). Regional THK retention also presented similar outcomes with global retention. Edges in resultant subnetwork were most connected to the parietal lobule, but some edges were connected within occipital and temporal regions. There were no significant correlations when using the positive range of threshold values. The other factors including amyloid, number of lacunes, and WMH volume had no significant relationship with connectivity disruption in EOAD.

**TABLE 2:** Cluster-based statistics (CBS) data assessing correlation between connectivity disruption and pathogenesis.

**Pair**		**# of clusters**	**# of edges in a cluster (*p*-value)**	**Representative regions**
EOAD	Global FLUTE SUVR	–	–	–
	Regional FLUTE SUVR	–	–	–
	Global THK SUVR	2	4 (0.0070), 3 (0.0136)	Precuneus (left and right)
	Regional THK SUVR	2	9 (0.0004), 5 (0.0008)	Middle occipital gyrus (right), superior parietal gyrus (left), Angular gyrus (left), and middle temporal gyrus (right)
	WMH volume	–	–	–
	Lacune	–	–	–
LOAD	Global FLUTE SUVR	1	9 (0.0288)	Middle occipital gyrus (left)
	Regional FLUTE SUVR	–	–	–
	Global THK SUVR	–	–	–
	Regional THK SUVR	–	–	–
	WMH volume	1	7 (0.0302)	Calcarine (left), and middle occipital gyrus (left)
	Lacune	1	10 (0.0354)	Middle occipital gyrus (left)

*CBS, cluster-based statistics; EOAD, early-onset Alzheimer’s disease; LOAD, late-onset Alzheimer’s disease; WMH, white matter hyperintensity.*

When considering LOAD, however, *W*-score networks had no significant correlation with THK retention. Using an identical threshold, LOAD had significant correlations in a negative direction with global FLUTE retention, number of lacunes, and WMH volume. As those pathologies became severe, the connectivity was damaged more in the identified edge sets. As shown in Table 2, all subnetworks derived in the LOAD group had one cluster. With global FLUTE retention, nine edges (*p* = 0.0288) comprised the identified subnetwork in which the left middle occipital gyrus was connected to the prevalent amount of edges. Either lacune numbers or WMH volume had the same representative region of the left middle occipital gyrus, as well as the left calcarine additionally in WMH volume analysis (Figure 3B). The common representative region indicates that the pathology-related connectivity disruptions in LOAD were mostly involved in the occipital lobule. Interestingly, there are only few overlapped edges among the resultant subnetworks even though there is moderate correlation between lacune numbers and WMH volume (Spearman ρ = 0.43). Just two edges concurred between global FLUTE retention and lacune numbers that do not have significant relations with each other, connecting the left middle occipital gyrus to the left fusiform gyrus and the left inferior occipital gyrus to the left inferior temporal gyrus. Global FLUTE retention was associated with disruption of relatively short edges connected to temporal or parietal regions, while vascular pathologies showed relationships with long connections to frontal or limbic areas. No factors were significantly correlated with the *W*-scores in a positive direction. For each AD group, pathological markers revealed disparate associations with spatially different disrupted connections.

### Association Between Factor-Related Connectivity Disruption and Cognitive Function

We extensively investigated relationships between those pathology-related edge disruptions and neuropsychological characteristics. We treated an averaged *W*-score of damaged edge sets concerned with each pathology as a representative value of each pathology-related network disruption. In EOAD, the averaged connectivity disruption related to global THK retention was significantly associated with all cognitive scores, except for some memory tests (Supplementary Table 2). Global cognition scores like MMSE and CDR SOB were also significantly associated with connectivity disruption. Scores in the domain of attention (digit span forward and backward), language (K-BNT), visuospatial (RCFT, copy), and executive function (COWAT, animal, supermarket and phonemic; Stroop test; TMT-A and B) had significantly positive correlations. Among the memory function tests, only SVLT immediate recall and recognition were associated with connectivity disruption. Regional THK retention was also associated with most cognitive functions. Several scores for attention and memory function showed weak correlations: digit span forward, SVLT immediate and delayed recall, and RCFT delayed recall.

On the contrary, LOAD showed fewer items with a significant relationship, and the correlation coefficients were also lower than in EOAD (Supplementary Table 2). MMSE (*r* = 0.4693, FDR-corrected *p* = 0.0233) or CDR SOB (*r* = −0.4618, FDR-corrected *p* = 0.0233) were associated with the edge weights having close relationships with global FLUTE retention. Of the specific cognitive domain tests, only digit span backward showed a significant association (*r* = 0.4803, FDR-corrected *p* = 0.0233). Individual pathology-related WM network disruptions did not seem to be associated necessarily with cognitive declines in LOAD compared to EOAD exhibiting significant correlations with various specific neuropsychological test scores.

## Discussion

In the present study, we observed that AD patients had significantly weaker WM structural connectivity compared to their age-matched control counterparts, and the patterns of disrupted connectivity were distinct between EOAD and LOAD. The topography of WM connectivity disruption was well correlated with the pathological and cognitive features of each AD group.

In the EOAD group, the most affected areas were identified in the frontal, parietal, and occipital lobes. The edges were located over all brain regions in EOAD patients, but connections linked with the parietal lobe comprised the largest proportion. These findings correlate with the pathological and clinical features of EOAD. In previous studies, EOAD patients have exhibited higher amyloid and tau burden in the parietal cortex (Ossenkoppele et al., 2012; Cho et al., 2017) and have shown widespread cortical atrophy especially in the parietal cortex (Noh et al., 2014a). In terms of clinical aspects, EOAD patients typically have poorer visuospatial function, traditionally viewed as a parietal function, compared to LOAD patients (Fujimori et al., 1998; Smits et al., 2012). Similar to EOAD, the frontal and parietal regions were also affected in LOAD patients. In LOAD, representative regions were additionally identified in the temporal and limbic lobes, and connection disruptions between the frontal and limbic lobes were remarkable compared to EOAD. These regions are primarily involved in memory function, which is mostly impaired in LOAD.

We further investigated which pathology was most associated with reductions in WM connectivity. *W*-scores represented the extent of reduction for all edges in our brain network. In EOAD patients, THK retention had a significant negative correlation with *W*-scores. This implies that there were several edges with significantly compromised connectivity related to growing THK burden. Both precuneus areas were the representative regions most associated with global THK retention. In a previous THK study with early AD patients, the precuneus was the most prominent AD-specific THK retention area, and the spatial pattern of THK uptake showed significant similarity with functional connectivity (Yokoi et al., 2018). The precuneus is functionally variable and plays an important role in the default mode network (DMN), which is known to be disrupted in EOAD and LOAD (Utevsky et al., 2014; Badhwar et al., 2017). Some previous studies have shown more pronounced DMN hypoconnectivity in EOAD compared to LOAD (Adriaanse et al., 2014; Thomas et al., 2014). The left superior parietal gyrus and angular gyrus were identified as the representative regions associated with regional THK retention in our study, as well as the right middle occipital and temporal gyrus. With THK retention, all and half of the representative regions were derived in the parietal lobule, and connections to the parietal or occipital lobules were found in common, consistently with previous researches reporting that greater tau burden exists in the parieto-occipital area with EOAD patients compared to LOAD (Cho et al., 2017). Interestingly, there were no correlations between amyloid or SVD markers and WM disruption. Tau might be the key factor for WM disruption in EOAD.

On the contrary, LOAD patients had quite different results. THK retention was not significantly associated with any edge declines in our network. In LOAD, global amyloid and SVD burdens were significantly associated with the connection disruptions between occipital and limbic or frontal regions. The occipital area was the common region associated with amyloid and SVD burdens. In a previous study, WMH were associated with amyloid burden especially in the occipital areas (Grimmer et al., 2012; Noh et al., 2014b). The posterior circulation may be vulnerable to injury of the endothelium leading to blood–brain barrier (BBB) disruption (Feske, 2011). BBB disruption may contribute to amyloid deposition through increased blockage of amyloid clearance (Zlokovic, 2011). Therefore, it is possible that SVD may result in preferential BBB disruption in the posterior circulation, which could induce increased amyloid deposition in these areas, including the occipital region (Noh et al., 2014b). There was also a small possibility of cerebral amyloid angiopathy (CAA). A previous study in CAA has demonstrated similar results with our study. In CAA patients, connectivity disruption was more pronounced in the occipital lobe and related with higher amyloid load and vascular markers including WMH volume (Reijmer et al., 2014).

Our findings show that EOAD and LOAD have distinct topographies and pathogenesis of WM connectivity disruption. Tau and amyloid pathology are considered to be critical biomarkers for the diagnosis of AD. Studies have reported that amyloid beta triggers the formation of toxic tau, and the toxic form of tau causes synaptic dysfunction and neuronal death (Hurtado et al., 2010; Bloom, 2014). Therefore, tau-related connectivity disruption in EOAD may be at least partly explained by this. In contrast, LOAD patients did not exhibit any tau-related findings. Only the global amyloid deposition and vascular markers were significantly related with connectivity disruption. Previous studies have shown that amyloid can cause neuronal dysfunction and cell death in the absence of tau pathology (Ramser et al., 2013; Bloom, 2014). Furthermore, unlike EOAD, development of LOAD may be affected by various factors including aging and vascular factors. The incidence of vascular pathology increases with age, and cerebral ischemia may be related with demyelination and axonal loss resulting in reduced WM connectivity.

Regarding the association with cognitive tests, tau-related WM disruption in EOAD was correlated with attention, language, visuospatial, frontal/executive functions, as well as MMSE and CDR SOB. Most weak correlations were shown in the memory domain, which was in line with previous studies (Smits et al., 2012; Cho et al., 2017). This sheds light on tau burden as a key influential factor and may imply that it mediates the distinct clinical patterns of EOAD. On the other hand, global amyloid-related WM disruption in LOAD was correlated only with a small number of the scores. This implies that the cognitive impairment in LOAD may be influenced by more complicated processes and multiple pathologies. Many recent studies about AD, mostly indicating LOAD, also reported that there are synergistic or mediated effects between pathological factors (e.g., tau and amyloid, or amyloid and vascular dysregulation) on AD progression (Iturria-Medina et al., 2016; Pascoal et al., 2017; Hanseeuw et al., 2019).

Several limitations must be considered for our findings. We did not include subcortical structures of medial temporal regions including the hippocampus or amygdala in our analyses, which could influence the results especially in LOAD patients. The deterministic tractography method for construction of white matter network could be another limitation, which is not able to consider crossing fibers completely. High-order models such as high angular resolution diffusion tensor (HARDI) (Tuch et al., 2002) and diffusion spectrum imaging (DSI) (Wedeen et al., 2005) can further resolve the crossing fiber issues, but the number of gradient directions we used was low to form enough degrees of freedom for those approaches. In addition, because it was shown that FA could be affected non-linearly by amyloid load in a previous study (Wolf et al., 2015), analyses regarding connectivity require caution. Finally, the current study investigated the association of various pathologies with heterogeneity between EOAD and LOAD using cross-sectional data, which could limit the interpretation of the results. Longitudinal analysis might be a possible direction for future works to further investigate causality between various pathologies and heterogeneity in the AD continuum.

In conclusion, we investigated the heterogeneous clinical features of EOAD and LOAD in terms of both Alzheimer’s and vascular pathology combining with the WM structural network. WM network disruption in both AD groups appeared to be distinct, with disruption in EOAD patients apparently affected by tau and related astrogliosis, which strongly correlated with most of the cognitive functions except for memory as being compatible with the general features of EOAD. LOAD patients showed associations with WM disruption with amyloid or vascular pathology; however, this disruption could not explain the cognitive profile of LOAD. The clinical features of LOAD might be influenced by more diverse factors and more complicated processes.

## Data Availability Statement

The raw data supporting the conclusions of this article will be made available by the authors, without undue reservation, to any qualified researcher.

## Ethics Statement

The studies involving human participants were reviewed and approved by the Institutional Review Board of Gachon University Gil Medical Center. The patients/participants provided their written informed consent to participate in this study.

## Author Contributions

J-KS, YN, and DN: conceptualization. WL and CY: writing-original draft. WL, S-WK, and HJ: data curation. WL, S-WK, and SS: formal analysis. J-KS and YN: funding acquisition. WL, SS, and J-KS: methodology. YN, DL, and J-KS: resources. WL, J-KS, YN, and SS: validation. J-KS and YN: writing-review and editing. All authors contributed to the article and approved the submitted version.

## Conflict of Interest

The authors declare that the research was conducted in the absence of any commercial or financial relationships that could be construed as a potential conflict of interest.

## Footnotes

1 http://www.fmrib.ox.ac.uk/fsl/

2 http://ccn.ucla.edu/wiki/index.php

## Abbreviations

AAL: automated anatomical labeling
AD: Alzheimer’s disease
APOE: apolipoprotein E
BBB: blood–brain barrier
CAA: cerebral amyloid angiopathy
CBS: cluster-based statistics
CDR: clinical dementia rating
CDR SOB: clinical dementia rating sum-of-boxes
CN: cognitively normal
CT: computed tomography
DMN: default mode network
DTI: diffusion tensor imaging
EOAD: early-onset Alzheimer’s disease
FA: fractional anisotropy
FDR: false discovery rate
FLAIR: fluid-attenuated inversion recovery
FLUTE: [^18^F]-Flutemetamol
LOAD: late-onset Alzheimer’s disease
MMSE: Mini-mental State Examination
MRI: magnetic resonance imaging
NBS: network-based statistics
OC: old controls
PET: positron emission tomography
ROI: region of interest
SUVR: standardized uptake value ratio
SVD: small vessel disease
THK: [^18^F]-THK5351
WM: white matter
WMH: white matter hyperintensities
YC: young controls.
