# Supplementary material for: Effects of Alzheimer’s and Vascular Pathologies on Structural Connectivity in Early- and Late-Onset Alzheimer’s Disease
Source: Front Neurosci. 2021 Feb 16;15:606600. doi: 10.3389/fnins.2021.606600 (PMC7921324; doi:10.3389/fnins.2021.606600)
Supplement: Supplementary file 1 [file Table_1.docx]

**Supplementary Table 1.** Neuropsychological test results

| Variables | EOAD  (n=50) | YC  (n=33) | EOAD vs YC p-value |  | LOAD  (n=38) | OC  (n=32) | LOAD vs OC  p-value |  | EOAD vs LOAD  p-value |
| --- | --- | --- | --- | --- | --- | --- | --- | --- | --- |
| Attention |  |  |  |  |  |  |  |  |  |
| Digit span forward | -0.48±1.36 | 0.73±0.93 | <0.001^*^ |  | 0.20±1.05 | 0.67±1.04 | 0.058 |  | 0.019^*^ |
| Digit span backward | -1.46±1.45 | 0.14±1.28 | <0.001^*^ |  | -0.37±1.14 | 0.09±1.10 | 0.085 |  | <0.001^*^ |
| Language and related function |  |  |  |  |  |  |  |  |  |
| K-BNT | -1.93±2.43 | 0.05±1.00 | <0.001^*^ |  | -1.63±1.41 | 0.06±0.89 | <0.001^*^ |  | 0.796 |
| Visuospatial function |  |  |  |  |  |  |  |  |  |
| RCFT copy | -5.42±5.76 | 0.58±0.57 | <0.001^*^ |  | -0.42±1.85 | 0.33±0.72 | 0.207 |  | <0.001^*^ |
| Memory |  |  |  |  |  |  |  |  |  |
| SVLT, immediate recall | -2.21±1.22 | -0.13±1.02 | <0.001^*^ |  | -1.45±0.84 | -0.07±0.85 | <0.001^*^ |  | 0.002^*^ |
| SVLT, delayed recall | -2.56±0.84 | -0.13±0.95 | <0.001^*^ |  | -1.96±0.58 | 0.11±0.97 | <0.001^*^ |  | <0.001^*^ |
| SVLT, recognition | -2.67±1.44 | -0.12±1.50 | <0.001^*^ |  | -1.42±1.27 | -0.02±0.85 | <0.001^*^ |  | <0.001^*^ |
| RCFT, immediate recall | -1.93±0.74 | 0.75±0.92 | <0.001^*^ |  | -1.20±0.76 | 0.34±1.12 | <0.001^*^ |  | <0.001^*^ |
| RCFT, delayed recall | -2.22±0.84 | 0.70±1.01 | <0.001^*^ |  | -1.40±0.80 | 0.42±0.98 | <0.001^*^ |  | <0.001^*^ |
| RCFT, recognition | -2.16±1.42 | 0.22±1.04 | <0.001^*^ |  | -1.71±1.67 | -0.22±0.95 | <0.001^*^ |  | 0.072 |
| Frontal executive function |  |  |  |  |  |  |  |  |  |
| COWAT, animal | -2.08±0.96 | -0.22±1.07 | <0.001^*^ |  | -1.62±0.88 | -0.26±1.05 | <0.001^*^ |  | 0.024^*^ |
| COWAT, supermarket | -1.75±0.92 | 0.05±0.94 | <0.001^*^ |  | -1.16±0.92 | 0.13±0.99 | <0.001^*^ |  | 0.015^*^ |
| COWAT, phonemic | -1.30±1.35 | 0.26±1.04 | <0.001^*^ |  | -0.87±0.91 | 0.15±1.06 | <0.001^*^ |  | 0.149 |
| Stroop test, color reading | -2.42±1.31 | 0.13±0.75 | <0.001^*^ |  | -0.93±0.93 | 0.17±1.12 | <0.001^*^ |  | <0.001^*^ |
| TMT-A | -7.27±11.1 | 0.59±0.77 | <0.001^*^ |  | -1.04±2.40 | -0.18±1.89 | 0.093 |  | <0.001^*^ |
| TMT-B | -8.40±6.23 | 0.01±1.00 | <0.001^*^ |  | -2.59±2.25 | -0.19±1.30 | <0.001^*^ |  | <0.001^*^ |

Data are presented as mean ± standard deviation of *Z*-scores

^*^ Significant

EOAD, early-onset Alzheimer disease; YC, young control; LOAD, late-onset Alzheimer disease; OC, old control; K-BNT, Korean version of the Boston Naming Test; RCFT, Rey-Osterrieth complex figure test; SVLT, Seoul verbal learning test; COWAT, controlled oral word association test; TMT-A, trail making test type A; TMT-B, trail making test type B
