# Supplementary material for: Effects of Alzheimer’s and Vascular Pathologies on Structural Connectivity in Early- and Late-Onset Alzheimer’s Disease
Source: Front Neurosci. 2021 Feb 16;15:606600. doi: 10.3389/fnins.2021.606600 (PMC7921324; doi:10.3389/fnins.2021.606600)
Supplement: Supplementary file 2 [file Table_2.DOCX]

**Supplementary Table 2.** Correlation between mean *W*-score within subnetwork from CBS and neuropsychological test scores

| Scores | EOAD | | LOAD | | |
| --- | --- | --- | --- | --- | --- |
|  | *Global THK SUVR* | *Regional THK SUVR* | *Global FLUTE SUVR* | *WMH volume* | *Lacune* |
| Digit Span Forward | 0.5795 (<0.001^*^) | 0.2421 (0.1071) | 0.1737 (0.5729) | 0.0380 (0.9585) | 0.0753 (0.9239) |
| Digit Span Backward | 0.7282 (<0.001^*^) | 0.4921 (0.0024^*^) | 0.4803 (0.0233^*^) | 0.0091 (0.9585) | 0.2247 (0.6996) |
| K-BNT | 0.5409 (<0.001^*^) | 0.3890 (0.0138^*^) | -0.0194 (0.9145) | 0.0097 (0.9585) | 0.0303 (0.9463) |
| RCFT Copy | 0.4560 (0.0019^*^) | 0.4865 (0.0024^*^) | 0.2645 (0.3208) | 0.0568 (0.9585) | 0.1475 (0.8082) |
| SVLT, immediate recall | 0.5881 (<0.001^*^) | 0.2457 (0.1038) | 0.3562 (0.1485) | 0.2680 (0.4975) | 0.0775 (0.9239) |
| SVLT, delayed recall | 0.1902 (0.2004) | 0.0928 (0.5350) | 0.1030 (0.7682) | 0.1176 (0.9022) | 0.1408 (0.8082) |
| SVLT, recognition | 0.4054 (0.0072^*^) | 0.3649 (0.0173^*^) | -0.0389 (0.8728) | 0.2114 (0.5015) | 0.0436 (0.9463) |
| RCFT, immediate recall | 0.2303 (0.1342) | 0.3842 (0.0138^*^) | -0.0493 (0.8728) | 0.0648 (0.9585) | 0.0118 (0.9463) |
| RCFT, delayed recall | 0.2044 (0.1781) | 0.2767 (0.0716) | -0.2156 (0.4803) | 0.0814 (0.9585) | -0.1614 (0.8082) |
| RCFT, recognition | 0.2945 (0.0534) | 0.4328 (0.0068^*^) | 0.0809 (0.7791) | 0.2734 (0.4975) | 0.1343 (0.8082) |
| COWAT, animal | 0.4661 (0.0018^*^) | 0.3619 (0.0173^*^) | 0.1277 (0.6995) | 0.2557 (0.4975) | 0.0492 (0.9463) |
| COWAT, supermarket | 0.4721 (0.0018^*^) | 0.4660 (0.0040^*^) | 0.2036 (0.5275) | 0.2396 (0.5015) | 0.1456 (0.8082) |
| COWAT, phonemic | 0.4774 (0.0018^*^) | 0.4334 (0.0068^*^) | 0.2853 (0.3208) | 0.2266 (0.5015) | 0.2915 (0.6524) |
| Stroop test, color reading | 0.5851 (<0.001^*^) | 0.5000 (0.0024^*^) | 0.1313 (0.6995) | 0.1413 (0.8658) | 0.0989 (0.9239) |
| TMT-A | 0.6556 (<0.001^*^) | 0.3698 (0.0173*) | 0.3531 (0.1708) | 0.0453 (0.9585) | 0.0245 (0.9463) |
| TMT-B | 0.3403 (0.0306^*^) | 0.3755 (0.0173^*^) | 0.0987 (0.7682) | 0.0291 (0.9585) | 0.2680 (0.6524) |
| MMSE | 0.6429 (<0.001^*^) | 0.4807 (0.0024^*^) | 0.4693 (0.0233^*^) | 0.3239 (0.4853) | 0.2633 (0.6524) |
| CDR SOB | -0.4523 (0.0018^*^) | -0.3836 (0.0134^*^) | -0.4618 (0.0233^*^) | -0.3989 (0.2359) | -0.3374 (0.6524) |

Neuropsychological test results are calculated as z-scores

Correlation coefficient (FDR-corrected p-value), ^*^ Significant

CBS, cluster-based statistics; EOAD, early-onset Alzheimer’s disease; LOAD, late-onset Alzheimer’s disease; WMH, white matter hyperintensity; K-BNT, Korean version of the Boston Naming Test; RCFT, Rey-Osterrieth complex figure test; SVLT, Seoul verbal learning test; COWAT, controlled oral word association test; TMT-A, trail making test type A; TMT-B, trail making test type B; MMSE, mini-mental state examination; CDR SOB, clinical dementia rating sum-of-boxes
